# Evidence- and opinion-based consensus from Delphi round 1 of the Asian aplastic anemia recommendation development

**DOI:** 10.1007/s12185-026-04240-z

**Published:** 2026-07-22

**Authors:** Jun Ho Jang, Yok Lam Kwong, Surapol Issaragrisil, Kohei Hosokawa, Ming Yao, Yeu-Chin Chen, Yeung-Chul Mun, F. K. Zhang, Lily Wong, Shinji Nakao

**Affiliations:** 1 Department of Hematology (Oncology), Samsung Medical Center, Seoul, South Korea; 2 The University of Hong Kong, Pok Fu Lam, Hong Kong; 3 Siriraj Hospital, Mahidol University, Salaya, Thailand; 4 Department of Hematology, Kanazawa University Hospital, Kanazawa, Japan; 5 Department of Internal Medicine, National Taiwan University, Taipei, Taiwan; 6 Division of Hematology/Oncology, Tri-Service General Hospital, National Defense Medical University, Taipei, Taiwan; 7 Department of Hematology, Ewha Womans University College of Medicine, Seoul, South Korea; 8 Anemia Therapeutic Center, Chinese Academy of Medical Science and Peking Union Medical College, Beijing, China; 9 Department of Hematology, Hospital Queen Elizabeth, Kota Kinabalu, Malaysia; 10 Department of Hematology, Kanazawa University, Kanazawa, Japan

**Keywords:** Aplastic anemia, Recommendation, Asia Pacific, Expert consensus, Delphi

## Abstract

**Background:**

Implementing international guidelines for aplastic anemia (AA) in Asia remains challenging due to limited access to advanced diagnostic tools, restricted treatment options, and variation in healthcare policies. This highlights a need for region-specific guidance on the diagnosis and management of AA in Asia.

**Methods:**

The Asian Aplastic Anemia Working Group consisted of 10 experts from across Asia (South Korea, Japan, China, Hong Kong, Malaysia, Taiwan, and Thailand) who met virtually twice and developed 16 thematic statements on the diagnosis and management of AA. Using an e-Delphi method, members voted asynchronously on these statements using a 5-point Likert scale. Consensus was predefined as “≥ 80% level of agreement among the Delphi participants.”

**Results:**

Sixteen thematic statements and 54 sub-statements were developed. The first three statements addressed AA definition, presentation, and causes; the next four focused on diagnosis and prognosis; and the remaining nine covered management, including clonal evolution, supportive care, and special populations. Fifteen statements reached consensus after the first voting round. However, one statement on the management of severe AA did not achieve consensus in the first round.

**Conclusion:**

This e-Delphi method has, for the first time, generated Asian, expert-derived, consensus recommendations for the diagnosis and management of AA.

**Supplementary Information:**

The online version contains supplementary material available at https://doi.org/10.1007/s12185-026-04240-z.

## Introduction

Aplastic anemia (AA) is a disorder of hematopoietic stem cells (HSCs) characterized by loss of blood cell precursors, hypoplasia or aplasia of the bone marrow, and cytopenia in ≥ 2 cell lines (red blood cells, white blood cells, and/or platelets) [1]. The incidence of severe AA (SAA) varies worldwide, ranging from 1.5 to 2.3 per million in Europe and North America to being 2–3 times higher (3.0–5.0 per million) in Asia (Table 1) [2–6]. Given the complexity of AA and the limited experience of healthcare practitioners, new guidelines for the diagnosis and management of adult AA were recently published by the British Committee for Standards in Haematology (BCSH) [7]. Compliance with certain aspects of international guidelines is challenging in the Asian setting, including implementing recommended therapies, a few of which are not available in Asia. For instance, the BCSH recommends horse antithymocyte globulin (ATG) in combination with cyclosporine A as the first-line immunosuppressive therapy (IST) regimen, but horse ATG is unavailable in many countries worldwide [7]. Rabbit ATG is used widely in Asia, with survival outcomes comparable to those with horse ATG [8]. Overall, uncertainty, underdiagnosis, and undertreatment prevail in AA management in Asia. These real-world limitations necessitate regionally adapted, pragmatic guidance.

**Table 1:** Incidence of aplastic anemia in Asia

Incidence of AA in Asia	
Country	Reported incidence
China	7.4 per million (1.4 per million for SAA and 6.0 per million for NSAA) [5, 6]
Thailand	4.6 per million (annual incidence of SAA/VSAA was 3.8 per million higher than that of NSAA, which was 0.8 per million, with a ratio of 4.6:1) [4]
Malaysia	4.8 per million
Taiwan	5.7 per million
Korea	5.16 per million [3]

AA, aplastic anemia; NSAA, non-severe AA; SAA, severe AA; VSAA, very severe AA

To address these challenges, a multidisciplinary Asian Aplastic Anemia Working Group was convened to adapt international evidence to the Asian context and produce consensus recommendations for adult AA. The Delphi method was used to develop and refine statements across investigation, initial therapy, and follow-up, with iterative rounds to achieve agreement thresholds. The aim of these expert consensus recommendations was to ensure that investigation, treatment, and monitoring decisions are based on the best available evidence and to promote and improve the best accepted practice based on the treatment options available in Asia. These recommendations are consensus-based and intended to support clinical judgment.

## Materials and methods

### Study design

The Asian Aplastic Anemia Working Group comprised a panel of international experts from across Asia (South Korea, China, Hong Kong, Japan, Malaysia, Taiwan, and Thailand). Their task was to develop guidelines for the diagnosis and management of AA in the routine Asian clinical context. The panel was created based on their clinical experience in managing AA and ability to evaluate relevant publications and translate the information to daily clinical practice. However, it was not feasible to base all recommendations purely on evidence due to scarcity of high-quality clinical trial evidence, and the experts relied on their own practical clinical experiences. Based on multiple rounds of deliberations and the Delphi process of voting, a set of 16 recommendation statements was developed for the Asia–Pacific (APAC) context. These statements represented the most ideal treatment paradigm for AA in the Asian context based on available therapies. The working group acknowledged that clinical decisions for individual patients may deviate from these recommendations and that this set of statements should not be considered a standard of care.

The methodology and steps undertaken to reach consensus on the recommendations are summarized in Supplementary Table 1. According to a study by Powell, the success of an investigation using the Delphi approach depends on the experts’ qualification, knowledge, experience, and expertise [9]. Ten experts were identified and invited to participate in the e‐Delphi study based on their wide clinical experience, expertise in AA, international standing, and employment in a variety of healthcare settings and across a breadth of Asian geographical locations.

### Compliance with ethics guidelines

As this study did not assess individual patient therapies or pathologies, did not collect sensitive personal or clinical data, and adhered to good data protection practices while posing minimal or no risk to participants, it was exempt from ethics committee review.

### Statistical analysis

Descriptive statistical analysis was conducted using SAS^®^ software V9.3 (SAS Institute, Cary, NC, USA). Percentages and mean scores are used to summarize the frequency of responses per Delphi round and for each statement. Continuous variables are described by number, mean, standard deviation, median, and range values. Categorical variables are described as the total number and relative percentage per category. Each statement was counted equally with no weighting applied to calculate consensus rates. Consensus was reached when ≥ 80% of the working group members scored a statement within the agreement thresholds (agree or strongly agree).

## Results

**Fig. 1:**
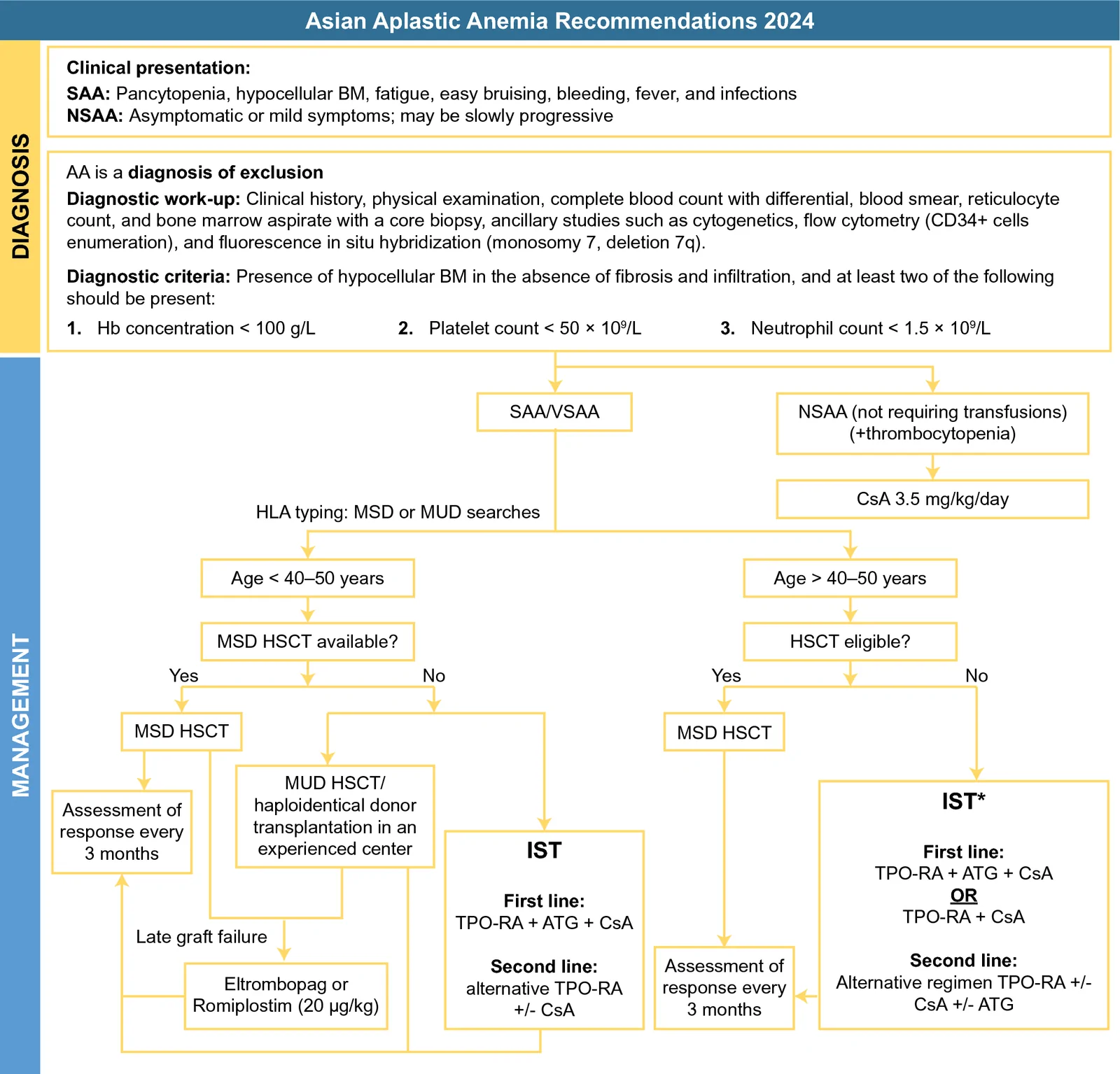
Algorithm for the diagnosis and management of AA in Asia *ATG can be skipped in very frail patients. AA, aplastic anemia; ATG, antithymocyte globulin; BM, bone marrow; CsA, cyclosporine A, Hb, hemoglobin; HLA, human leukocyte antigen; HSCT, hematopoietic stem cell transplantation; IST, immunosuppressive therapy; MSD, matched sibling donor; MUD, matched unrelated donor; NSAA, non-severe aplastic anemia; SAA, severe aplastic anemia; TPO-RA, thrombopoietin agonist; VSAA, very severe aplastic anemia

Sixteen thematic statements, including 54 sub-statements, were developed to address the diagnosis and management of AA. With a two-round e-Delphi method, 15 out of 16 statements reached the predefined consensus threshold of ≥ 80% agreement in the first round, while one statement required a second round but ultimately did not achieve consensus. The finalized recommendations covered definitions, diagnostic criteria, severity assessment, treatment strategies, and supportive care, with detailed voting outcomes summarized in supplementary tables. The algorithm for diagnosis and management of AA in Asia is shown in Fig. 1. All consensus-based thematic statements on AA are presented in Table 2, while the percentage of agreement (with standard deviation) for each statement is presented in Supplementary Table 2.

**Table 2:** Consensus-based thematic statements on AA from the Asian Aplastic Anemia Working Group

	Statement	Detail
1	Definition	AA is a bone marrow failure disorder characterized by pancytopenia with hypocellular bone marrow in the absence of abnormal infiltration, marrow fibrosis, or increased reticulin
2	Clinical presentation	AA presents with pancytopenia and hypocellular bone marrow, along with symptoms of fatigue, easy bruising, or bleeding and/or fever or infections in the absence of a long-standing illness. The common sites of bleeding are the skin, nose, and gums. NSAA can be asymptomatic, present with mild symptoms, and progress slowly
3	Etiology	The etiology of AA can be idiopathic, an IBMFS, or linked to environmental or occupational toxins such as benzene, viral infections, contaminated water sources, medication (e.g., chloramphenicol and anti-epileptics), herbal medicines, and exposure to animal fertilizers and agricultural pesticides. There is no evidence supporting a higher incidence of hepatitis-associated AA in Asia. Most cases of acquired AA are idiopathic
4	Diagnosis	(a) AA is considered a diagnosis of exclusion, and the initial diagnostic workup focuses on distinguishing AA from other diverse causes of cytopenias (b) Diagnosis of AA is based on clinical history, physical examination, and complete blood count with differential, blood smear evaluation, reticulocyte count, and bone marrow aspirate with a core biopsy, with ancillary studies such as cytogenetics, flow cytometry (CD34^+^ cell enumeration), and fluorescence in situ hybridization (monosomy 7, deletion 7q) (c) Macrocytosis and normochromicity are the major features of red cell morphology in the chronic form, while normocytosis and normochromicity are common in the acute form (d) Screening for somatic mutations in bone marrow cells using NGS is not necessary for the diagnosis of and therapeutic planning for AA, but screening for germline mutations is useful for ruling out IBMFS. Somatic mutation analysis may help to identify susceptibility to acute myeloid leukemia/myelodysplastic syndromes and is useful in monitoring the natural progression of the disease, including clonal evolution, and supporting clinical research programs. Currently, the treatment plan at diagnosis remains unchanged if somatic mutations are identified using NGS
5	Diagnostic criteria	AA can be diagnosed by the presence of hypocellular bone marrow in the absence of fibrosis and infiltration, with at least two of the following findings: (a) Hemoglobin concentration < 100 g/L (b) Platelet count < 50 × 10^9^/L (c) Neutrophil count < 1.5 × 10^9^/L
6	Severity assessment	The Camitta criteria were used to assess the severity of AA (a) SAA: Marrow cellularity < 25% (or 25%–50% with < 30% residual hematopoietic cells) plus at least two of the following: neutrophil concentration < 0.5 × 10^9^/L; platelet count < 20 × 10^9^/L; and absolute reticulocyte count < 60 × 10^9^/L (b) VSAA: Same as for SAA, but with neutrophil concentration < 0.2 × 10^9^/L (c) NSAA: AA not fulfilling the criteria for SAA or VSAA
7	Prognosis	Older age, severe disease, and longer duration from diagnosis to treatment are poor prognostic predictors in AA
8	Aims of treatment	The aims of AA treatment are as follows: (a) To manage symptoms related to anemia and thrombocytopenia (b) To prevent serious infections and hemorrhage (c) To achieve transfusion independence by restoring hematopoiesis
9	Assessment of response to treatment	Complete response: meeting all of the following criteria: (a) Platelet count ≥ 100 × 10^−9^/L (b) ANC ≥ 1.0 × 10^−9^/L (c) Hb level ≥ 100 g/L Partial response: (a) Blood counts that no longer meet the criteria for severe AA but also do not meet the criteria for complete response (b) No requirement for blood transfusion Assessment is performed after 3 and 6 months of treatment
10	Transplant-based management	(a) Choice of modality: i. SAA and VSAA require immediate treatment as the interval between diagnosis and treatment is a strong predictor of survival ii. The first treatment modality for patients with SAA/VSAA aged < 40–50 years is HLA-MSD HSCT iii. IST can be the first choice of therapy for patients who do not have an HLA-matched donor, those older than 40–50 years, or those who are ineligible for allo-HSCT iv. Haploidentical HSCT can be considered in SAA/VSAA because the outcome in experienced centers is equivalent to those of MSD and MUD HSCT v. HLA-MUD HSCT can be recommended to young patients without a matched sibling (b) Workup: i. All patients who are potential allo-HSCT candidates should undergo HLA typing at diagnosis, followed by MSD or MUD searches, as appropriate, to assess the availability of potential donors. Further, the selection of family donors without inherited mutations is important ii. It is also imperative to not miss constitutional AA to avoid serious toxicity from the conditioning regimen (c) Key considerations: The decision regarding HSCT depends on resources, infrastructure, availability of donors, and cultural aspects (d) Late graft failure: Allo-HSCT recipients with complete or partial donor chimerism who experience late graft failure may be treated with the TPO-RAs eltrombopag and romiplostim, before considering a second allo-HSCT
11	Treatment of SAA/VSAA	(a) TPO-RAs: i. Both romiplostim and eltrombopag are suitable TPO-RAs in the first-line treatment of SAA/VSAA ii. TPO-RA dosage for refractory AA: The doses of romiplostim and eltrombopag effective for SAA/VSAA are much higher than those used for immune thrombocytopenia. For romiplostim, the starting dose is 10 µg/kg/week and is increased in increments of 5 µg/kg/week until a maximum dose of 20 µg/kg/week. The preferred starting dose of eltrombopag is at least 50–75 mg, and it is increased in increments of 25–50 mg, if reticulocyte or platelet response is not observed, up to a maximum dose of 150 mg/day. Note that in the Asia–Pacific region, individual drug dosages may vary according to regional policies iii. Treatment duration: If reticulocyte or platelet response is not observed within 3–6 months of initiating treatment, the patient is less likely to respond further. In such cases, the patient may be switched to another TPO-RA. If the patient responds well, treatment is continued until hematologic response peaks and is then tapered off iv. Eltrombopag leads to gastrointestinal side effects (dyspepsia and gastritis). It may also cause transaminitis. High-dose eltrombopag may cause skin pigmentation and may also interfere with bilirubin interpretation, following which the laboratory must be informed (b) ATG: Response rates with rabbit ATG in Asian patients with AA are similar to those with horse ATG reported in Western countries. However, comparative studies are needed to confirm that rabbit ATG is as effective as horse ATG (c) Additional considerations for treatment: i. Patient characteristics, including duration of the disease, pathophysiology, presence/absence of immune markers, and age, should be considered before initiating combination therapy ii. Supportive measures alone, growth factors, androgens, cyclosporine A, or corticosteroids alone are not definitive therapies for SAA and are inferior in efficacy compared with conventional immunosuppression regimens iii. Anabolic steroids can be used as an alternative therapy for patients who cannot afford or are not suitable for allo-HSCT or IST. Their long-term use, particularly in female patients, must be carefully considered and monitored (d) First-line treatment: i. “TPO-RA + ATG + cyclosporine A” is preferred ii. In elderly patients with frailty, the combinations “TPO-RA + ATG + cyclosporine A” and “TPO-RA + cyclosporine A” are both equally preferred iii. ATG can be skipped in very frail patients due to safety issues (e) Second-line treatment: i. For patients who received “TPO-RA + ATG + cyclosporine A” as the first-line therapy, the second-line therapy should include an “alternative TPO-RA” + / − cyclosporine A ii. For patients who received “TPO-RA + cyclosporine A” as the first-line therapy, the second-line therapy should include an “alternative TPO-RA” + / − cyclosporine A + / − ATG iii. The combination of therapies is dependent on patient characteristics, including duration of disease, pathophysiology, and presence/absence of immune markers
12	Treatment of NSAA	Patients with NSAA not requiring transfusions should be treated with cyclosporine A monotherapy (3.5 mg/kg/day), particularly if thrombocytopenia is observed
13	Concern of clonal evolution	(a) Romiplostim and eltrombopag are not as commonly associated with clonal evolution in Asian patients (incidence of only about 5%), and this reduced risk compared with that in Western patients (regardless of receiving or not receiving TPO-RAs) might be attributable to a very low prevalence of the HLA-B*14:02 allele in Asian patients (b) Serial bone marrow testing to monitor clonal evolution is not recommended outside of clinical trials when a patient’s AA shows improvement in response to therapy, except for patients who present with abnormal karyotype/NGS data
14	Treatment failure	(a) HSCT from an HLA-matched alternative donor is usually the second-line treatment for patients who experienced IST failure (b) Switching to the other TPO-RA is recommended when treatment failure with one TPO-RA is observed. Cyclosporine A can be continued in combination with TPO-RA (c) A second course of ATG may be indicated following failure to respond to the first course if the patient is not an HSCT candidate
15	Supportive care	(a) Almost all patients required supportive care, which includes blood transfusion, antibiotics, hematopoietic growth factors, and iron chelation therapy (b) Blood transfusions should be performed to improve quality of life. The hemoglobin concentration threshold should be individualized (c) Prophylactic platelet transfusions should be administered to patients with stable AA receiving active treatment, and a platelet count threshold (pretransfusion) of 5–10 × 10^9^/L should be used (d) Implement regular long-term follow-up to monitor hematologic parameters and to detect relapse and subsequently clonal disorders such as myelodysplastic neoplasms, leukemia, paroxysmal nocturnal hemoglobinuria, and solid tumors (e) Emphasize the importance of psychosocial support and counseling for patients and their families to cope with the chronic nature of AA and its treatment
16	Special Population	(a) Pregnancy: i. Supportive care is the mainstay of treatment for AA in pregnancy, aiming to maintain the platelet count above 20 × 10^9^/L with platelet transfusions ii. Low-dose cyclosporine A is considered safe for use in pregnancy, if needed (b) Elderly: i. IST in combination with TPO-RAs is the treatment of choice in the elderly as in younger patients: ATG and cyclosporine A treatment result in a more rapid recovery of blood counts and, alternatively, cyclosporine A alone or anabolic steroids with TPO-RAs can be considered

AA, aplastic anemia; allo-HSCT; allogeneic hematopoietic stem cell transplantation; ANC, absolute neutrophil count; ATG, antithymocyte globulin; CD, cluster of differentiation; Hb, hemoglobin; HLA, human leukocyte antigen; HSCT, hematopoietic stem cell transplantation; IBMFS, inherited bone marrow failure syndrome; IST, immunosuppressive therapy; MSD, matched sibling donor; MUD, matched unrelated donor; NGS, next-generation sequencing; NSAA, non-severe AA; SAA, severe AA; TPO-RA, thrombopoietin receptor agonists; VSAA, very severe AA

## Discussion

### Statement 1. Definition

The Manual of Pediatric Hematology and Oncology (2011) defines AA as “a physiologic and anatomic failure of the bone marrow characterized by a marked decrease or absence of blood-forming elements in the marrow and pancytopenia (decreased red cells, white blood cells and platelets)” [10]. The currently proposed definition is adapted from the British Society for Haematology (BSH) guidelines [7, 11]. The working group members unanimously agreed to this definition. The clinical course of AA varies from mild to severe, and the progression can be either slow or aggressive. Acquired AA, also called idiopathic AA, represents a majority (∽ 70%) of all newly diagnosed AA cases [12, 13]. Acquired AA will hereafter be referred to as “AA” in this report.

### Statement 2. Clinical presentation

Patients may present along a spectrum of clinical manifestations, ranging from being asymptomatic with incidental findings on peripheral blood testing to having life-threatening neutropenic infections or bleeding. The clinical presentation of patients with AA can be categorized into symptoms related to a decrease in bone marrow production of hematopoietic cells such as the following:

- Anemia (pallor, headache, palpitations, dyspnea, fatigue, or foot swelling)
- Neutropenia (frequent and persistent minor infections, sudden-onset febrile illness, overt infections, recurrent infections, or mouth and pharyngeal ulcerations)
- Thrombocytopenia (ecchymoses, mucosal and gingival bleeding, or petechial hemorrhages) [14].

Notably, splenomegaly is not seen in AA, and its presence suggests an alternative diagnosis. Laboratory findings include macrocytic normochromic anemia with reticulocytopenia, neutropenia, and thrombocytopenia. Cytologic abnormalities should be absent as their presence would suggest an alternative underlying hematologic condition. Based on their clinical experience, the experts commented that, although uncommon, a few patients with SAA often experience a preceding non-severe AA (NSAA) phase over a period of several years. The experts also echoed the need to consider the definitions of cell counts that fall within the range of severe AA.

### Statement 3. Etiology

AA currently, by definition, refers to acquired AA. This excludes the etiology of inherited bone marrow failure syndromes (IBMFS) resulting from loss-of-function germline mutations. The experts agreed that IBMFS should not be referred to as AA to be consistent with the next recommendation for diagnosis. Regarding the correlation with hepatitis, the experts opined that there was no evidence supporting a higher incidence of hepatitis-associated AA in Asia.

Direct marrow damage is the cause of a small proportion of AA and could be due to exposure to chemical agents or irradiation. Benzene was a common causative agent worldwide for bone marrow failure, with an early dose-dependent effect of exposure. Incidence of AA due to benzene has decreased in countries where occupational exposure is regulated.

Most of the cases labeled as idiopathic AA are currently considered to be due to immune-mediated insults to the hematopoietic system. A reduction in the efficacy of regulatory T cells (Treg cells) is associated with AA [15–17]. Somatic mutations have also been detected in AA [18–20].

### Statement 4. Diagnosis

The diagnosis of AA is challenging due to varied presentations and overlapping conditions. The absence of a single definitive test makes it a diagnosis of exclusion [21]. Accurate diagnosis requires thorough history taking, physical examination, and comprehensive clinical, hematologic, and subcellular assessments.

The diagnostic approach can be divided into five steps [21, 22]:

#### (1) Establishing the presence of cytopenia (peripheral counts, peripheral smear, and reticulocyte counts)

Hemoglobin concentrations, neutrophil counts, and platelet counts are uniformly depressed in AA. Isolated thrombocytopenia may occur in the early stages of AA. The BSH guidelines stress the importance of a peripheral smear in the diagnosis of AA. It is crucial to exclude the presence of abnormal cells such as dysplastic neutrophils, abnormal platelets, blasts, and peculiar cells such as “hairy cells.” The appearance of the smear can further help to distinguish between acute and chronic presentations of the disease (see step 4).

#### (2) Excluding other causes of cytopenia

Liver function tests can detect ongoing hepatitis. Viral studies can be performed to rule out an infectious cause of AA. Testing for antinuclear antibodies and anti-double-stranded DNA can be performed to rule out pancytopenia along with autoimmune conditions including systemic lupus erythematosus, which may be associated with myelofibrosis, or rarely hypocellular bone marrow.

Abdominal ultrasound can reveal enlarged spleen or lymph nodes that may suggest a malignant hematologic disorder. Displaced kidneys are suggestive of Fanconi’s anemia. A low reticulocyte count superimposed on pancytopenia usually suggests bone marrow pathology as the likely cause of the low counts.

#### (3) Establishing whether the bone marrow is the primary site responsible for the cytopenia (bone marrow aspirate and core biopsy)

A bone marrow aspirate confirms hypocellularity. Difficulty in obtaining particles may indicate infiltrative diseases. Dysplastic changes are typically not observed, but erythroid dysplasia, with or without mild myeloid dysplasia, is common in patients with the chronic form of AA. Dysplastic megakaryocytes should not be observed.

A non-tangential core/trephine biopsy measuring ≥ 1.5 cm is necessary to confirm aspirate findings, meet the minimum definition of AA (statement 1), and rule out abnormal infiltration of the marrow (leukemia, solid tumors, storage disorders) and increased marrow fibrosis (myelofibrosis).

#### (4) Ruling out other causes of bone marrow failure (flow cytometry, immunohistochemistry, and genetic testing)

Hypoplastic myelodysplastic neoplasms (MDS), paroxysmal nocturnal hemoglobinuria (PNH), and IBMFS also present with pancytopenia in the background of hypocellular bone marrow. Thus, they might be mistaken for AA. Once bone marrow failure has been established with hypocellular bone marrow, these other causes must be ruled out.

Presence of monocytopenia increases the likelihood of IBMFS. Genetic testing, preferably using next-generation sequencing on panel-based assays, can identify mutations associated with known IBMFS [23]. They can also provide evidence of clonal evolution. Cytogenetic abnormalities, while considered characteristic of MDS, may transiently appear in otherwise typical AA [24, 25]. Diagnostic algorithms that combine phenotypic, laboratory, and genetic data are now being employed in research settings and increasingly in the clinical settings.

Flow cytometric detection of PNH clones and their quantification using appropriate biomarkers help to differentiate between individuals with AA and those with PNH causing aplasia [26].

Hypoplastic MDS remains a challenging differential diagnosis. Immunohistochemistry for markers such as TP53, CD34, and CD117 may be useful in differentiating between hypoplastic MDS and AA [27].

#### (5) Identifying variants and rare etiologies of AA (emerging diagnostic tests)

A careful review of history can detect exposure to an offending drug or toxin that may have precipitated bone marrow failure. Emerging diagnostic tests not yet used in routine clinical practice can further clarify etiology and exclude other conditions. Telomere shortening in peripheral blood leukocytes is observed in dyskeratosis congenita (DC) and can also occur in acquired AA with low bone marrow reserves [28]. Studying gene panels for telomere complex mutations is useful for ruling out other IBMFS. Somatic mutation analysis can be a useful monitoring tool to indicate the likelihood of progression to MDS/acute myeloid leukemia (AML), although it does not impact treatment [29]. Single-nucleotide polymorphism array karyotyping can be used to detect unbalanced chromosomal defects [30].

### Statement 5. Diagnostic criteria

The current recommendations retain the original diagnostic criteria proposed by Camitta et al. [31]. The experts noted that thrombocytopenia is more pronounced than other cytopenias at the time of diagnosis in cases of non-SAA (NSAA). Although not mentioned in the BSH guidelines, thrombocytopenia is an important feature of AA. Hence, thrombocytopenia is a prerequisite for the diagnosis of AA. In the presence of normal platelet counts, the diagnosis of AA based on anemia and neutropenia must be made with extreme caution.

### Statement 6. Severity assessment

Severity stratification of AA is useful in deciding the prognosis and management plan. In addition to severity, response to initial therapy, disease duration, and patient age are also factors that determine prognosis.

The modified Camitta criteria for AA severity stratification are based on bone marrow cellularity and peripheral counts [30, 32]. They have been widely accepted and are used in the BSH guidelines.

For younger patients with SAA/very SAA (VSAA), the treatment approach is comparatively aggressive. In contrast, for patients with NSAA, the approach is more conservative and supportive in nature. Severity must be assessed not only during diagnosis but also during ongoing treatment as the patient may progress from NSAA to SAA. Neutropenia has the greatest impact on reducing survival compared with the other two components of the Camitta criteria. Further, many patients with an absolute neutrophil count (ANC) below 0.1 × 10^9^/L present with septic conditions, have a shorter survival, and are unable to undergo aggressive treatment or receive hematopoietic stem cell transplantation (HSCT) or IST [33].

### Statement 7. Prognosis

Older age, high Charlson comorbidity index at baseline, and VSAA have been found to be independently associated with mortality [34]. A hierarchy of prognostic factors had been proposed in the following order: concurrent severe infections, ANC, percentage of non-myeloid cells on bone marrow slides, platelet count, reticulocyte count, ^59^Fe utilization, and stromal disorganization observed in bone marrow biopsy specimens [35]. In an analysis of 105 patients with AA, the most significant variable impacting prognosis at diagnosis was reticulocyte count, followed by the percentage of bone marrow lymphocytes, red cell turnover rate, T-score severity grade, iron utilization, and Aplastic Anemia Study Group (AASG) severity grade. Reticulocyte count was the only significant variable in another multivariate analysis [36]. An ANC of < 0.5 × 10^9^/L also has a significantly negative impact on overall survival (OS) in AA [37]. A Chinese study reported that age of 36–65 years, SAA/VSAA, and no specific therapy were independent risk factors for inferior survival [38].

### Statement 8. Aims of treatment

The aim of management is to reduce, and ideally eliminate, the need for transfusions and restore protection from infections [39]. Some studies have defined the goals of immunosuppression as improved life expectancy and a durable hematologic response that prevents relapse and clonal evolution [40]. Two treatment aims have been proposed to be important, viz. supportive management to correct the initial symptoms and treatment of bone marrow failure. A hospital clinical management framework proposed aims of AA management to include improvement of clinical AA recognition, timely diagnosis, completion of pretreatment laboratory evaluations, initiation of treatment, improvement of patient outcomes by standardizing assessment of treatment response, and initiation of second-line treatment when indicated [41]. From the perspective of patients, the main goals of management are to alleviate SAA symptoms such as chronic fatigue, infection risk, and bleeding risk due to low platelet counts.

### Statement 9. Assessment of response to treatment

A robust treatment response has been correlated with better OS [42]. Defining treatment response is essential for timely initiation of next-line therapy if the previous regimen is not considered effective. The BSH guidelines used the response criteria reported by Marsh et al. (2009) [11, 43].

In most clinical trials, hematologic response is assessed at 3-month and 6-month intervals. The experts suggested that in routine clinical practice, the frequency of assessment may be set to a 3-month schedule. Clinical studies have used absolute reticulocyte count, ANC, and absolute lymphocyte count as potential response predictors [44].

### Statement 10. Transplant-based management

Allogeneic HSCT (allo-HSCT) is a definitive treatment for AA, but IST and stem cell stimulation can be adjuncts or substitutes (see Statement 11). Allo-HSCT can be performed using a human leukocyte antigen (HLA)-matched or haploidentical donor. An HLA-matched donor can be a matched sibling donor (MSD) or a matched unrelated donor (MUD). Unavailability of suitable donors and transplantation-related complications have limited the success rate of HSCT. Outcomes are better with the use of bone marrow rather than peripheral blood HSCs [45, 46], antithymocyte incorporation for conditioning, younger donor age [47], and a shorter interval from diagnosis to treatment [48, 49].

The risk of complications has been stratified by patient and donor characteristics. With sibling donors, younger patients have better outcomes and fewer complications. In contrast to the 50% long-term survival in adults aged ≤ 40 years [50], the survival rate is above 80% in adolescents and is even higher at 90% in young children [51].

HSCT from unrelated histocompatible donors does not impact survival rates but results in a twofold higher rate of graft-versus-host disease. Late complications are also more frequent with unrelated donor HSCT [49, 52].

There is variability in the clinical paths of patients who respond to IST [53]. Cell counts may not recover to normal levels, long-term cyclosporine A is required to prevent relapse [54], and late consequences such as clonal evolution with cytogenetic abnormalities and the development of MDS or AML may occur [22]. All patients receiving ATG require high standards of care and monitoring in an experienced in-patient setting.

Availability of suitable donors varies geographically. Several studies [55–58], including a large multicenter study from China [59], suggest that using T cell-depleting strategies before and after HSCT is highly beneficial.

The experts noted that excellent results from recent studies suggest that haploidentical HSCT could be used as a first-line treatment for fulminant AA, which is characterized by a critically low neutrophil count, even after a week of granulocyte colony-stimulating factor (G-CSF) therapy [60]. Moreover, it could be considered before MUD HSCT in VSAA. In other cases of AA, including VSAA, IST should be considered before attempting haploidentical HSCT.

MUD HSCT is no longer considered inferior to MSD in children [48, 61]. A multicenter British study found that children who failed other therapies and received unrelated grafts had 95% survival [62]. The experts suggested that allo-HSCT from HLA-matched unrelated donors should be the first-line treatment for pediatric patients only. The decision to use a MUD for patients aged 20–40 years should be made clinically in consultation with the patient based on multiple factors such as severity of AA and fertility considerations.

### Statement 11. Treatment of SAA/VSAA

IST can be used as a first- or second-line therapy. A combination of ATG and cyclosporine A leads to a hematologic response in two-thirds of patients [63]. Prednisolone is added to prevent the side effects of ATG [64].

Retrospective Asian data have shown horse and rabbit ATGs to be equally efficacious, whereas horse ATG has been recommended for first-line IST in the BSH guidelines [7, 43].

Stem cell stimulation was not very successful until the advent of oral thrombopoietin-receptor agonists (TPO-RAs). Eltrombopag was initially licensed for chronic idiopathic thrombocytopenic purpura [65]. However, it has shown robust durable activity in patients with refractory AA, with half of the patients showing improvement in all three lineages [66, 67] and responses sustained even after drug discontinuation in some patients. Addition of eltrombopag to IST increased response rates further to approximately 80% [68]. The rates of relapse and clonal evolution were similar in patients treated with IST alone and those treated with IST and eltrombopag. The preferred dose of eltrombopag is at least 50–75 mg for refractory cases and can be increased up to 75 mg when used in combination with ATG and cyclosporine A. However, it should be noted that in Japan and South Korea, the approved maximum dosage is 100 mg/day [69].

Romiplostim has shown a similar efficacy and safety profile in patients with refractory AA [70, 71]. Romiplostim has also been reported to achieve a response rate of 84% at 27 weeks, with a trilineage response of 39% at 53 weeks [70]. In September 2023, romiplostim was approved in Japan for first-line use [72, 73]. Thus, a combination of TPO-RA (either eltrombopag or romiplostim) with ATG and cyclosporine A is recommended as the first-line treatment in patients who are not candidates for allo-HSCT. Subsequent publications reported the efficacy of romiplostim combined with CsA or with ATG plus CsA in adult patients with AA [72, 73]. An open-label, phase 2/3 study demonstrated the efficacy and safety of CsA combined with romiplostim in patients previously untreated with IST, with an overall response rate of 42% at 27 weeks [72]. In another open-label, phase 2/3 study, romiplostim combined with ATG and CsA demonstrated efficacy in adult patients with AA previously untreated with IST, with overall response rates of 41% and 77% at weeks 14 and 27, respectively [73]. A study reported an increase in mortality in patients aged > 60 years who received ATG [74]. The experts suggested that ATG can be avoided in frail elderly patients, who may instead be treated with cyclosporine A plus TPO-RA [72, 75]. Elderly patients with SAA should be individually assessed for frailty, considering their specific wishes.

Some patients may respond to TPO-RA monotherapy, particularly those with NSAA. Thus, in practice, any combination of ATG, cyclosporine A, and/or TPO-RA may be given to an individual patient based on patient characteristics and disease markers. Non-response to one TPO-RA does not rule out the potential for response to the other one.

The experts recommended that “TPO-RA for aplastic anemia treatment can be started at a relatively large dose and quickly increased to the maximum dose, as there is no need to be concerned about thrombosis. Starting with small doses may not be effective, with gradual dose escalation not being time-efficient.” They also noted that in some countries (e.g., Japan), insurance companies do not approve rapid dose increases.

As timely treatment determines the outcome, a lack of response within 3–6 months should trigger a switch to an alternative TPO-RA. The experts suggested that setting the lower limit at 3 months for the observation period is advisable, as it helps shorten treatment time.

### Statement 12. Treatment of NSAA

The BSH guidelines recommend treating patients with NSAA if they have transfusion dependence or neutropenia [7]. The experts suggested that cyclosporine A monotherapy has good outcomes, and the results of a prospective study in Japan demonstrated that a low-dose cyclosporine A therapy produced a platelet response in 31% of 32 patients at 16 weeks [76]. Japanese guidelines for the treatment of NSAA not requiring transfusions recommend cyclosporine A (3.5 mg/kg/day) monotherapy, particularly when thrombocytopenia is evident [77]. The guidelines state, “For patients with stage 1 and stage 2a AA, which is defined as mild AA not requiring transfusions, initiating cyclosporine A as early as possible after the diagnosis is recommended.”

Data also support the use of thrombopoietin mimetics in NSAA. Initial research suggested that eltrombopag in combination with cyclosporine A was effective [78], and based on the safety profile and efficacy of TPO-RA in refractory AA, it might also be a promising treatment option for treatment-naïve NSAA.

In a study of 95 Chinese pediatric patients with NSAA, cyclosporine A monotherapy showed heterogeneous effects [79]. The study concluded that treatment approaches in NSAA should be hierarchical and individualized. Patients with lower platelet counts and higher interleukin 10 levels were more likely to respond to cyclosporine A. The study had subcategorized patients as having mild NSAA, moderate NSAA, and transfusion-dependent NSAA.

In a Japanese retrospective analysis of 42 patients with NSAA, transfusion-free survival of patients with advanced-stage NSAA was significantly prolonged with IST (p = 0.002) [80]. In addition, a recent, large multicentric study of 238 patients with NSAA showed that cyclosporine A + / − eltrombopag led to a significant clinical improvement in NSAA, with a favorable impact on survival [81].

### Statement 13. Clonal evolution

Improvement in long-term survival of patients with AA has led to the possibility that clonal evolution may occur, which may progress to MDS and AML. Next-generation sequencing data showed that clonal genetic changes might develop in AA [20, 82], with the most common being the development of PNH clones [83, 84], followed by the somatic loss of HLA loci [85, 86]. Both changes indicated the occurrence of immune escape [87] and were associated with improved response to IST [88, 89]. Targeted sequencing of genes implicated in hematologic malignancies has revealed somatic mutations in MDS-associated genes in up to one-third of patients with AA [29, 90].

However, only about 10% of patients show cytogenetic abnormalities. Of these, monosomy 7/del 7q is associated with a poor prognosis, with a worse response to IST and an increased progression to MDS. In contrast, trisomy 8 and del (13q) are associated with an improved response to IST and a better prognosis [82].

Overall, up to 15% of patients with AA treated with IST may develop MDS or AML in Western countries [91, 92]. Until now, limited data suggest that the incidence of clonal evolution is not higher in patients treated with TPO-RA [93–95].

In Asian patients treated with TPO-RA, the incidence of clonal evolution appears to be lower than that in Western patients receiving similar regimens or IST. Furthermore, progression to MDS is rare [95, 96]. However, for patients without karyotypic aberrations but with genetic mutations at presentation, the experts do not recommend serial bone marrow testing to monitor clonal evolution.

### Statement 14. Treatment failure

Overall, the evidence for second-line unrelated donor HSCT after failed IST has improved but still remains scarce [97]. A prospective multicenter study compared the efficacy of repeated IST with HSCT from an alternative donor in children with acquired SAA who failed to respond to an initial course of IST [98]. After 5 years, OS was similar between the two treatment groups. However, failure-free survival was significantly higher in the allo-HSCT group, suggesting possible additional benefits. In another retrospective analysis of 49 patients with SAA, post-HSCT outcome was comparable between patients who underwent upfront allo-HSCT and those who underwent HSCT after IST failure [99].

### Statement 15. Supportive care

Survival of patients with AA after IST or allo-HSCT has improved substantially over the past decades. For patients who received IST, even the prognosis of non-responders to initial IST has substantially improved over time. This improvement is largely attributable to improved supportive care.

Supportive care in AA is centered around the prevention and early treatment of fungal, bacterial, and viral infections; transfusion strategy; and iron chelation therapy. Careful management of hemorrhage, bacterial and fungal infections, and secondary events such as alloimmunization to blood products and iron overload can considerably impact the prognosis of patients with AA.

Best supportive care is beneficial at all stages of AA, starting from initial presentation. In SAA, the quality of best supportive care often determines survival. It should be continued together with management strategies, whether with allo-HSCT or ATG. Because response to ATG may take up to 3 months or longer, best supportive care is critical during this time to help ensure an optimal outcome.

A critical component of supportive care is transfusion of red cells and platelets, very occasionally white cells. Hematopoietic growth factors, prophylactic antibiotics, and antifungals may also be used in special cases.

During treatment with ATG, worsening thrombocytopenia may occur due to increased platelet consumption in the presence of cross-reacting antibodies present in ATG binding to platelets. This may create a need for regular platelet transfusion support. For patients with thrombocytopenia requiring invasive procedures, platelet transfusions must be administered, aiming to achieve a platelet count in line with the BSH guidelines for the relevant procedures [100]. Although there are no studies to support the exact threshold for platelet transfusion support prior to ATG, most authors use a threshold of 20 × 10^9^/L [40].

The use of irradiated granulocytes should be considered in patients with life-threatening infections related to severe neutropenia, which anecdotally may be lifesaving [101]. Prophylactic antifungal therapy with voriconazole or posaconazole is also used in individuals with ANC < 500 × 10^9^/L. This is extrapolated from better survival after their use post-HSCT in patients with acute leukemia [102].

The target hemoglobin concentration may vary in patients with AA depending on symptoms. The optimal use of red blood cells involves the administration of enough red blood cells to maximize the clinical outcome while avoiding unnecessary transfusions [103]. A restrictive transfusion policy is ideal, but for patients who are elderly or have comorbidities (especially underlying coronary heart disease), the target hemoglobin concentration may vary from 8 to 10 g/dL [104]. Blood preparations from siblings or a family donor should be avoided to minimize the risks of developing transfusion-related graft-versus-host disease and graft failure if these donors are used for allo-HSCT, owing to immune response to donor-specific antigens [105]. Leukocyte-depleted and irradiated products (dose, 25–30 Gy) are used to reduce, among other risks, the risk of febrile post-transfusion reactions, alloimmunity, and cytomegalovirus infection [106].

In very elderly patients or those with moderate pancytopenia, supportive care may be the only therapy used [107]. For candidates of allo-HSCT, transfusions should be used selectively to reduce the risk of immunization to donor antigens.

### Statement 16. Special population

It is possible for women with AA to conceive, as fertility is not affected. AA during pregnancy is associated with significant fetal, neonatal, and maternal morbidity and mortality [108]. The fetus may experience growth retardation or neonatal sepsis [109]. The incidence of obstetric and neonatal complications ranges between 12 and 33% [110, 111]. If thrombocytopenia is present, the incidence of hemorrhagic complications during the peripartum period might be as high as 75% [112].

Allo-HSCT is obviously not feasible during pregnancy [113]. Supportive management with transfusions that aims for a hemoglobin concentration of > 8 g/dL and platelet count of > 10–20 × 10^9^/L is recommended [114]. There are reports of successful treatment of pregnant patients with prednisolone, cyclosporine A, and G-CSF [115]. There is limited published evidence around the use of ATG during pregnancy.

Transfusion of blood products is the mainstay of supportive treatment in AA during pregnancy, but carries the risk of complications, including hemochromatosis and HLA alloimmunization [116]. Alloantibodies against human platelets cause platelet transfusion refractoriness. The benefit of transfusions in preventing bleeding should be weighed against the likelihood of developing alloimmunization and hemochromatosis in the mother [79].

If corticosteroids are used, those unable to cross the placenta, such as prednisone, prednisolone, and hydrocortisone, should be preferred to prevent fetal brain exposure and the albeit small risk of orofacial malformations [117].

The use of G-CSF has been shown to be a safe and effective therapy and may be recommended when AA is accompanied by significant neutropenia [74].

### Strengths and limitations of these recommendations

These recommendations have several strengths and limitations, as listed in Supplementary Table 3.

### Future prospects

New treatment evidence, and hence paradigms, may emerge together with novel therapeutic modalities. Therefore, revisions of these recommendations are expected in the future.

## Conclusions

AA is a complex disorder requiring a comprehensive, multidisciplinary management approach. Evidence, resource, time, and reimbursement constraints, together with limited APAC-specific guidelines, limit the standardized and optimized management of AA in the Asian setting. This e-Delphi method has, for the first time, generated Asian, expert-derived, consensus recommendations for the diagnosis and management of AA. It is envisaged that the evidence-based guidance and expert opinion provided in this article can support the optimization of AA management in the APAC region and potentially in other regions at large.

## Supplementary Information

Below is the link to the electronic supplementary material.

Supplementary file1 (DOCX 35 KB)

## Data Availability

Not applicable.
