# Supplementary material for: Evidence- and opinion-based consensus from Delphi round 1 of the Asian aplastic anemia recommendation development
Source: Int J Hematol. 2026 Jul 22;124(4):521–37. doi: 10.1007/s12185-026-04240-z (PMC13638747; doi:10.1007/s12185-026-04240-z)
Supplement: Supplementary file 1 [file 12185_2026_4240_MOESM1_ESM.docx]

**Supplementary Material**

**Supplementary Table 1** Methodology followed for the development of the Asian Aplastic Anemia Consensus

| **No.** | **Steps** | **Timeline** |
| --- | --- | --- |
| 1 | The need for clinical guidelines pertinent to the Asian context was identified by an advisory board. | December 2022 |
| 2 | The group was reconvened to form the Asian Aplastic Anemia Working Group. |  |
| 3 | Key clinical questions were circulated to all working group experts for review to ensure that all relevant areas of clinical practice were covered. |  |
| 4 | Sixteen thematic statements addressing the clinical questions were proposed. |  |
| 5 | The 16 statements were discussed at length during a 3-h virtual meeting. | June 2023 |
| 6 | The statements were further subjected to review via email correspondence. |  |
| 7 | **E-Delphi Voting Round 1**  The e‐Delphi survey was shared with the Asian Aplastic Anemia Working Group via the online platform e-Delphi (<https://www.edelphi.org/index.page>). Background information was shared via email, with verbal consent obtained thereafter. The experts were sent unique URL links via the e-Delphi platform and were asked to rate their level of agreement on a 5-point Likert scale (1 meaning strongly disagree and 5 meaning strongly agree) and comment elaborately on the rationales behind their responses. The participants were requested to complete the survey within 2 weeks. An additional 1-week extension was granted after a reminder email. On completion of the survey, the median scores and agreement levels were calculated for each statement. The percentage of participant consensus and median risk score were calculated. | July 2023 |
| 8 | All statements except statement 11 received consensus in the first round of voting. |  |
| 9 | **E-Delphi Voting Round 2**  Statement 11 was subjected to a second round of voting after rephrasing based on the working group’s comments. | October to December 2023 |
| 10 | The statements were finalized. | December 2023 |
| Other aspects | Consensus was defined as “≥80% level of agreement among the Delphi participants.” The organizers discontinued the rounds if consensus was not reached after two rounds. The statements were excluded from the following rounds if a consensus was reached or if a minimum of 50% of consensus was not reached in the first round. | |

**Supplementary Table 2** Consensus achieved on each of the statements of the Asian Aplastic Anemia Recommendations

| **Statement No.** | **Topic** | **Agreement (%)** | **Consensus achieved** | **Round when consensus was achieved** | **Standard deviation** |
| --- | --- | --- | --- | --- | --- |
| **1** | Definition | 100 | Yes | First | 0 |
| **2** | Clinical presentation | 100 | Yes | First | 0.4 |
| **3** | Etiology | 90 | Yes | First | 0.831 |
| **4** | Diagnosis | 100 | Yes | First | 0.4 |
| **5** | Diagnostic criteria | 100 | Yes | First | 0 |
| **6** | Severity assessment | 100 | Yes | First | 0.4 |
| **7** | Prognosis | 100 | Yes | First | 0.458 |
| **8** | Aims of treatment | 100 | Yes | First | 0.4 |
| **9** | Assessment of response to treatment | 80 | Yes | First | 0.4 |
| **10** | Treatment modality | 100 | Yes | First | 0 |
| **11** | Treatment of severe aplastic anemia/very severe aplastic anemia | (Ongoing)* | Yes | Second | (Ongoing) |
| **12** | Treatment of non-severe aplastic anemia | 80 | Yes | First | 0.6 |
| **13** | Concern about clonal evolution | 90 | Yes | First | 0.3 |
| **14** | Treatment failure | 100 | Yes | First | 0.4 |
| **15** | Supportive care | 100 | Yes | First | 0 |
| **16** | Special population | 100 | Yes | First | 0 |

* The agreement was noted as ongoing as romiplostim clinical trial results had not yet been published as of the meeting date. These results were published at a later date [1, 2].

**Supplementary Table 3** Strengths and limitations of the recommendation statements

| **Strengths** | **Limitations** |
| --- | --- |
| 1. The Delphi methodology allowed active participation of the therapeutic area experts involved in the analysis process of a clinical pathway that is often challenging and supported by insufficient scientific evidence. 2. In constituting the working group, highly skilled and experienced healthcare professionals who manage AA on a routine basis collaborated. 3. The predefined strict criterion of “≥80% agreement” strengthened the results. After the second Delphi round, all but one of the statements reached the prefixed cutoff. This high level of consensus in the first round was the result of pre-work and deliberation with the experts prior to circulating the statements for voting. 4. There was a 0% attrition rate and 100% retention rate throughout the two rounds of the e-Delphi approach. | 1. The Delphi method was limited by the small sample size. However, there is no gold standard for calculating the sample size for Delphi panels. In published studies, the panel size varies from 10 to 1,000 members. As mentioned in the Materials and Methods section, the success of an investigation using the Delphi approach results from the “qualification of the experts,” rather than their quantity. Moreover, several Delphi studies have been conducted with <15 panelists [3]. Furthermore, the strict eligibility criteria led to the inclusion of high-profile professionals. 2. Only Asian experts were included. Global policies have greatly influenced therapeutic choices for AA management in Asia, and the addition of a global expert would have added a broader perspective. However, the experts from Asia were all of international standing, but their clinical practice was based in Asia. 3. Health economics and drug costs were not assessed when developing these recommendations; when more than one option is clinically appropriate, cost may reasonably influence choice. 4. This does not account for variability in the regulatory approval rules and insurance reimbursement policies across all participating countries |

**References**

1. Lee JW, Jang JH, Yamazaki H, Sawa M, Kizaki M, Tomiyama Y et al. Addition of romiplostim to immunosuppressive therapy as first-line treatment for patients with aplastic anemia. Blood Adv. 2025;9(22):5732–35. doi:10.1182/bloodadvances.2024014855.
2. Lee JW, Jang JH, Chiba S, Yoon SS, Oshikawa G, Usuki K et al. Romiplostim with ciclosporin A in patients with aplastic anaemia naïve to immunosuppressive therapy: a phase 2/3 study. Br J Haematol. 2025;207(2):582–90. doi:10.1111/bjh.20206.
3. McPherson S, Reese C, Wendler MC. Methodology update: Delphi studies. Nurs Res. 2018;67(5):404–10. doi:10.1097/nnr.0000000000000297.
